# Spectrum and clinical profile of disorders of sex development: A 10 year experience at a tertiary care center

**DOI:** 10.12669/pjms.42.9.15195

**Published:** 2026-09

**Authors:** Nida Aslam, Sommayya Aftab, Bushra Naseer Khan, Noor-e-Hira

**Affiliations:** 1 Dr. Nida Aslam, (FCPS Pediatrics Medicine) Fellow Pediatrics Endocrinology and Diabetes, The Children’s Hospital and University of Child Health Sciences, Lahore, Pakistan; 2 Sommayya Aftab, (FCPS Pediatrics Medicine, MRCPCH, FRCPCH), The Children’s Hospital and University of Child Health Sciences, Lahore, Pakistan; 3 Bushra Naseer Khan (FCPS Pediatrics Medicine) Fellow Pediatrics Endocrinology and Diabetes, The Children’s Hospital and University of Child Health Sciences, Lahore, Pakistan; 4 Noor-e-Hira (MBBS), The Children’s Hospital and University of Child Health Sciences, Lahore, Pakistan

**Keywords:** 46XY DSD, 46XX Turner syndrome, Ambigous genitalia, Disorders of Sex Development, DSD, Gonadal Dysgenesis, Karyotyping

## Abstract

**Background & Objective:**

Disorders of Sex Development is a group of congenital conditions characterized by atypical development of chromosomal, gonadal, or anatomical sex. These conditions present diverse clinical challenges and require individualized, multidisciplinary management throughout life. This study aimed to determine the clinical spectrum of children and adolescents with Disorders of Sex Development (DSD) at a tertiary care center over the last decade.

**Methodology:**

In this retrospective observational study, 815 children and adolescents (0–18 years) diagnosed with Disorders of Sex Development (DSD) were evaluated at the Department of Pediatrics Endocrinology and Diabetes, University of Child Health Sciences, The Children’s Hospital, Lahore from January 2015 to December 2024. Data regarding demographic characteristics, clinical presentation, karyotype, and final diagnosis were collected and analyzed. Statistical analysis was performed to assess the association between karyotype and subtype distribution, with significance set at p < 0.05.

**Results:**

The mean age of presentation was 7.5 ± 5 years, with 62% raised as male and 38% as female. The most common presentation was microphallus (41%), followed by ambiguous genitalia (21.6%) and delayed puberty/primary amenorrhea (20%). Based on karyotype, 46,XY DSD was most common (60.7%), predominantly due to androgen insensitivity and 5α-reductase deficiency (82.4%). 46,XX DSD accounted for 33.4%, mainly congenital adrenal hyperplasia (91.6%), while sex chromosome DSD comprised 6.6%, with Turner Syndrome variants being the most frequent subtype. A statistically significant association was observed between karyotype and subtype distribution (χ² = 167.42, p < 0.0001).

**Conclusion:**

DSD presents a broad range of clinical challenges underscoring the need for early accurate diagnosis and appropriate gender assignment. A multidisciplinary approach, improved diagnostic tools, and better education are essential for achieving optimal patient outcomes and reducing the long-term psychological impact of DSD.

## INTRODUCTION

Disorders of sex development (DSD) are a group of congenital conditions in which there is atypical development of chromosomal, gonadal, or anatomical sex. Although these conditions are rare, variations in definitions over time have led to differences in how they are reported and studied. The current terminology was introduced in 2005 by a joint consensus of the Lawson Wilkins Pediatric Endocrine Society and the European Society for Paediatric Endocrinology.^1^ According to this classification, DSD is divided into three main groups: 46,XX DSD (related to ovarian development or excess androgen exposure), 46, XY DSD (related to testicular development or androgen synthesis/action), and sex chromosome DSD, which includes conditions such as Turner syndrome, Klinefelter syndrome, mixed gonadal dysgenesis, and ovotesticular DSD.^2^

The epidemiology of DSD is not completely clear, mainly because of differences in definitions and reporting. The overall incidence is estimated to be between 1 in 3,000 and 1 in 5,500 live births.^3^ Among specific causes, congenital adrenal hyperplasia (CAH) is one of the most common, with a prevalence of around one in 13,000 to 18,000 births. Atypical genitalia in 46,XX newborns has been reported in approximately one in 30,000 to 45,000 births.^4^

Clinically, DSD can present at different ages. Some patients present at birth with ambiguous genitalia, while others may present later with delayed puberty, primary amenorrhea, or infertility.^5^ The causes are varied and include genetic abnormalities as well as environmental and maternal factors, such as drug exposure during pregnancy. Evaluation requires a stepwise approach including clinical assessment, karyotyping, hormonal evaluation, and imaging to assess internal reproductive structures. In selected cases, laparoscopy or gonadal biopsy may be required.^6^ Management usually involves a multidisciplinary team, including endocrinologists, surgeons, and psychologists, to address both medical and psychosocial aspects of care.^7^

Despite advances in diagnosis and management, data from resource-limited settings remain limited. Most published studies come from high-income countries, where access to genetic testing and specialized care is more readily available. In contrast, in low- and middle-income settings, factors such as delayed presentation, limited diagnostic facilities, and sociocultural influences on gender assignment can significantly affect patient outcomes. These factors are not well represented in the existing literature. Understanding these differences is important for developing practical and locally relevant management strategies.

This study aimed to evaluate the clinical spectrum and karyotype distribution of DSD in children presenting to a tertiary care center over the past decade. It also aims to identify gaps in diagnosis and management in a resource-limited setting, with the goal of improving early recognition and multidisciplinary care.

## METHODOLOGY

This retrospective observational study enrolled 815 children and adolescents (mean age 7.5 ± five years, range 0–18 years) diagnosed with Disorders of Sex Development (DSD) at the Department of Paediatric Endocrinology and Diabetes, University of Child Health Sciences, The Children’s Hospital, Lahore, from January 2015 to December 2024.

### Ethical approval:

This study was performed in accordance with the Declaration of Helsinki. This human study was approved by IRB of The Children’s Hospital and University of Child Health Sciences Lahore, Pakistan - approval: March 04, 2025; Reference number 1082/CH-UCHS. All parents, guardians or next of kin provided written informed consent for the minors to participate in this study.

###  Inclusion Criteria:

All patients with Diagnosis confirmed by using clinical features, biochemical tests, hormonal profiles, radiological findings (Ultrasound pelvis, MRI pelvis), and karyotyping(46XX ,46XY,other chromosomal abnormalities) were included. In some cases, additional investigations including gonadal biopsy, laparoscopy, and hormone stimulation tests were done. Predefined clinical criteria suggestive of disorders of sexual development, adapted from established international guidelines. These criteria were used to ensure systematic identification of suspected DSD cases presenting to our center.

### Exclusion Criteria:

Patients were excluded if they had incomplete clinical records, lack of essential diagnostic workup (including hormonal or chromosomal analysis), or conditions mimicking DSD without true disorders of sexual differentiation. Additionally, patients who were lost to follow-up before a definitive diagnosis could be established were excluded to maintain the reliability and validity of outcome assessment.

## RESULTS

At presentation, 38% (n=310) were living as female and 62% (n=505) as male. The most common presenting complaint was microphallus (41%, n=334), followed by ambiguous genitalia (21.6%, n=176), hypospadias with undescended testes (11%, n=90), delayed puberty or primary amenorrhea (20%, n=163), and virilization at puberty (6.4%, n=52). Early presentations was often due to adrenal crisis, leading to a congenital adrenal hyperplasia (CAH) diagnosis, while late presentations in males were frequently due to referrals from other specialties for DSD workup. In females delayed presentation was mostly due to the concern of short stature, virilization ,hirsutism and delayed puberty, Fig.1.

**Fig.1:**
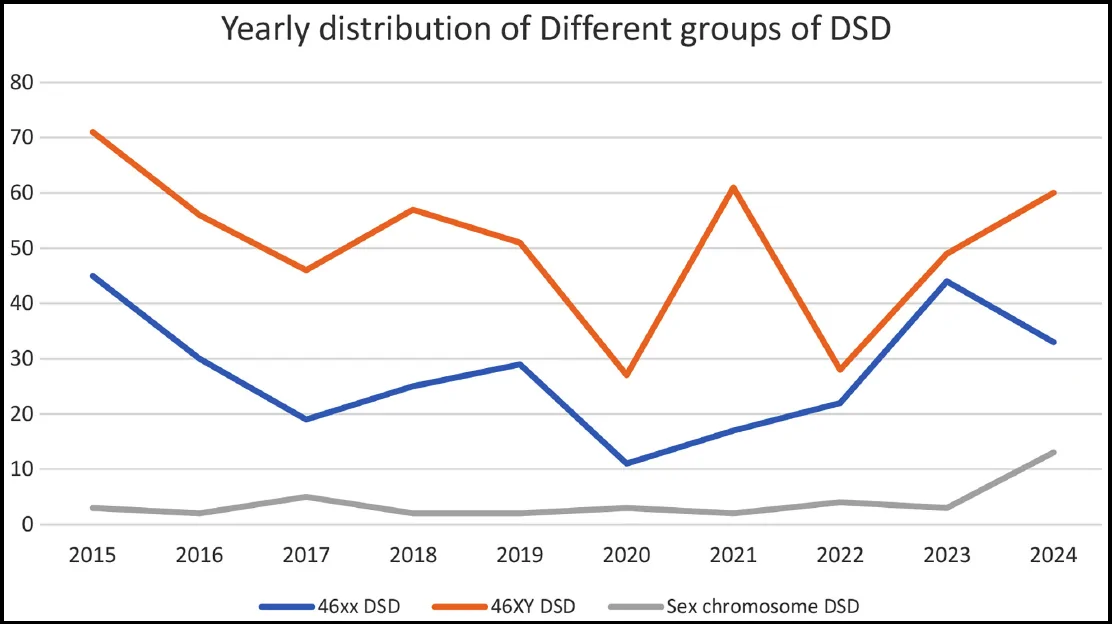
Yearly distribution of different groups of DSD.

**Fig.2:**
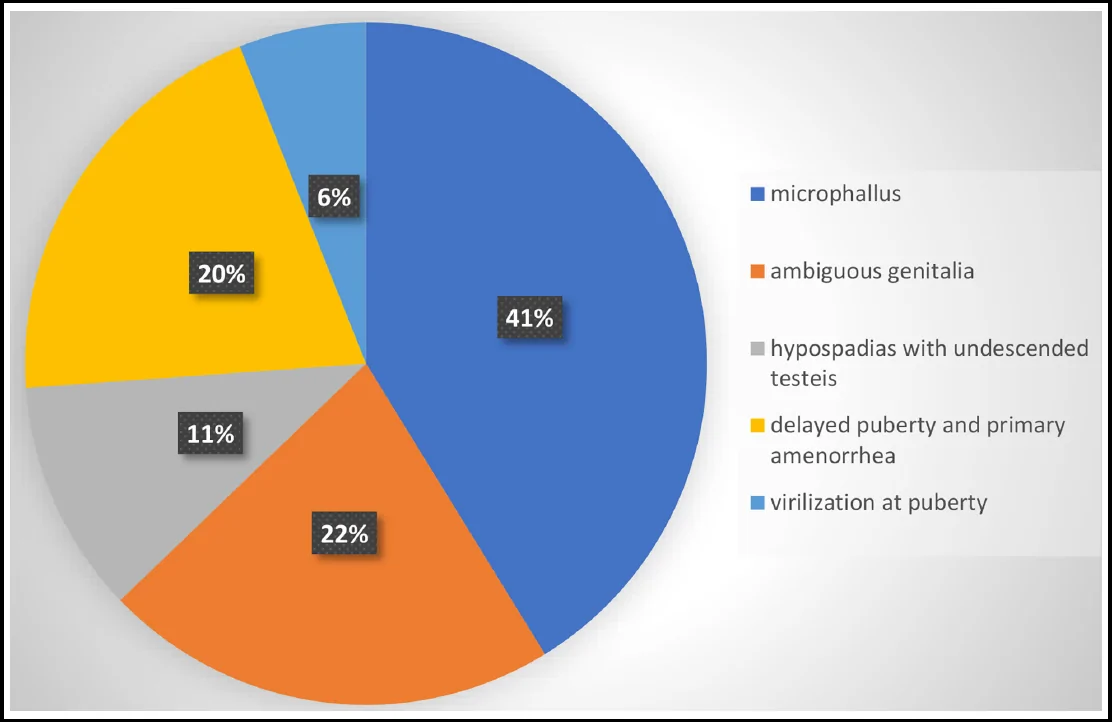
Common presenting complaints.

DSD diagnosis was categorized by karyotype: 46,XY DSD (60.7%, n=495), 46,XX DSD (33.4%, n=275), and Sex Chromosome DSD (6.6%, n=54). A chi-square test of independence revealed a significant association between karyotype and subtype distribution (χ² = 167.42, df = 4, p < 0.0001), indicating distinct subtype patterns across karyotypes .

### 46,XY DSD:

The most prevalent category was 46,XY DSD (60.7%, n=495), with Partial/Complete Androgen Insensitivity Syndrome or 5α-Reductase Deficiency (PAIS/CAIS/5αR) dominating (82.4%, n=408). Other subtypes included 46,XY Gonadal Dysgenesis (6.9%, n=34), Ovotesticular DSD (3.8%, n=19), CAH (3.4%, n=17), Gonadal Regression Syndrome (2.2%, n=11), Isolated Hypospadias (0.8%, n=4), Persistent Mullerian Duct Syndrome (0.2%, n=1), and LH Receptor Mutation (0.2%, n=1).

### 46,XX DSD:

46,XX DSD accounted for 33.4% (n=275) of cases, predominantly CAH (91.6%, n=252). Less frequent subtypes were Ovotesticular DSD (3.3%, n=9), Isolated Clitoromegaly (1.8%, n=5), Aromatase Deficiency (1.5%, n=4), 46,XX Testicular DSD (1.1%, n=3), and 46,XX Gonadal Dysgenesis (0.7%, n=2).

### Sex Chromosome DSD:

Sex Chromosome DSD comprised 6.6% (n=54) of cases, with 43 cases identified as Turner Syndrome variants, including Monosomy 45,XO (42.6%, n=23) and Mosaic 45,XO/XX (14.8%, n=8). Other subtypes were Mixed Gonadal Dysgenesis (25.9%, n=14) and Klinefelter Syndrome (16.7%, n=9), Table-II.

**Table-I:** Clinical Criteria Used for Inclusion of Patients with Suspected DSD.

Category	Clinical Features Suggesting DSD
Genital Ambiguity at Birth	- Overt genital ambiguity (genitalia not clearly male or female)
Apparent Female Genitalia	- Enlarged clitoris (clitoromegaly)
	- Posterior labial fusion
	- Inguinal/labial mass
Apparent Male Genitalia	- Bilateral undescended testes (cryptorchidism)
	- Micropenis (stretched penile length <2.5 cm at birth in term newborn)
	- Isolated perineal hypospadias
	- Mild hypospadias with undescended testis/testes
Pubertal Abnormalities	- Delayed or absent puberty
	- Undervirilization in individuals with 46,XY karyotype
Karyotype Discordance	- Discrepancy between genital appearance and prenatal karyotype (e.g., 46,XX with virilized genitalia or 46,XY with female-appearing genitalia)
Associated Features	- Lymphedema in the newborn period
	- Short stature or learning difficulties in childhood

**Table-II:** Distribution of DSD Subtypes by Karyotype (n=815)

Karyotype	Major Subtype 1	Major Subtype 2	Other Subtypes	Total
46,XX DSD	CAH: 252 (91.6%)	N/A: 0 (0%)	23 (8.4%)	275 (33.4%)
46,XY DSD	PAIS/CAIS/5αR: 408 (82.4%)	46,XY Gonadal Dysgenesis: 34 (6.9%)	53 (10.7%)	495 (60.1%)
Sex Chromosome DSD	45,XO: 23 (42.6%)	Mixed Gonadal Dysgenesis: 14 (25.9%)	17 (31.5%)	54 (6.6%)
Total	683 (83.8%)	48 (5.9%)	84 (10.3%)	815 (100%)

## DISCUSSION

This cross-sectional study characterizes the clinical spectrum, karyotypic distribution, and subtype prevalence of disorders of sexual development (DSD) in a large pediatric cohort from a resource-limited setting, offering a population-specific perspective that remains underrepresented in the global literature. The observed predominance of 46,XY DSD (60.7%), with Partial/Complete Androgen Insensitivity Syndrome and 5α-Reductase Deficiency (PAIS/CAIS/5αRD) accounting for 82.4% of this subgroup, is consistent with regional data reporting elevated rates of undervirilization in 46,XY individuals, likely amplified by the high consanguinity rates characteristic of our population.^8^,^9^ The statistically significant association between karyotype and subtype distribution (χ² = 167.42, df = 4, p < 0.0001) validates the current DSD classification framework 46,XY, 46,XX, and sex chromosome DSD and underscores the distinct etiological pathways inherent to each category.^10^

The predominance of Congenital Adrenal Hyperplasia (CAH) as the leading cause of 46,XX DSD (91.6%) reflects a well-established epidemiological pattern in high-consanguinity populations, where autosomal recessive 21-hydroxylase deficiency is disproportionately prevalent.^11^ Critically, a subset of patients presented following sibling deaths from undiagnosed adrenal crises a sentinel finding that highlights the life-threatening consequences of absent neonatal screening programs in our setting. This observation provides a compelling, population-specific argument for implementing universal newborn screening for classical CAH, as even a single prevented neonatal death justifies programmatic investment in resource-constrained healthcare systems. The frequent presentation of micropenis (41%), ambiguous genitalia (21.6%), and hypospadias with cryptorchidism (11%) emphasizes the key role of standardized neonatal physical examination as a primary diagnostic approach, particularly in resource-limited settings.^12^,^13^

Late presentations manifesting as delayed puberty and primary amenorrhea in 20% of cases represent a critical diagnostic gap attributable to both limited specialist access and insufficient clinician awareness of DSD phenotypes.^14^ This pattern is especially pronounced in 46,XY DSD males, who are frequently referred late due to initial misclassification or cultural reluctance to investigate ambiguous phenotypes, a pattern documented in regional Pakistani cohorts.^15^ Notably, 62% of patients in our cohort were raised as male despite ambiguous genitalia or undervirilization a proportion substantially higher than reported in Western cohorts where sex assignment follows structured multidisciplinary, patient-centered protocols.^16^ This divergence suggests that cultural preferences for male gender assignment in South Asian populations may override clinical guidance,^17^,^18^ This may have important long-term effects on gender identity development, which needs to be studied further through long-term follow-up.^19^ The high prevalence of androgen pathway defects in 46,XY DSD, particularly 5α-reductase deficiency, highlights the need for targeted *SRD5A2* mutation analysis. Identifying population-specific variants could enable development of cost-effective, locally relevant genetic testing and support earlier diagnosis, providing a pathway for integrating genomic medicine into DSD care in resource-limited settings.^10^,^20^

The inclusion of sex chromosome DSD (6.6%), encompassing Turner Syndrome variants (Monosomy 45,X: 42.6%; Mosaic 45,X/XX: 14.8%) and Klinefelter Syndrome (16.7%), reflects the broadened contemporary classification of DSD as a spectrum incorporating both gonadal and phenotypic variability.^20^ Rare subtypes such as Persistent Müllerian Duct Syndrome and LH Receptor Mutation (each 0.2%) are likely underrepresented, reflecting the constraints of a single-center study and limited access to confirmatory molecular genetic testing.^20^ In our cohort Turner Syndrome presented mostly with short stature and delayed puberty, along-with additional dysmorphic features necessitating systematic screening for associated anomalies, consistent with established management guidelines.^21^ A novel observation in this study is the high burden of associated systemic anomalies including cardiac, renal, and autoimmune disorders in Turner Syndrome patients, which has historically gone undocumented in our region due to the absence of structured registries. Enrollment in international frameworks such as the I-DSD Registry represents an urgent priority to systematically capture this comorbidity data and enable comparative epidemiological analysis.^22^ The significant proportion of mixed gonadal dysgenesis (25.9%) underscores the diagnostic complexity of this category, which frequently necessitated advanced investigations including gonadal biopsy and diagnostic laparoscopy in our cohort.^23^

Ovotesticular DSD was identified in both 46,XY (3.8%) and 46,XX (3.3%) individuals, confirming that this rare subtype exists and requires comprehensive clinical ,biochemical ,radiological and histological evaluation of gonads beyond karyotyping alone, consistent with recommended diagnostic frameworks for ambiguous genitalia.^23^ This finding highlights an important research gap. Rare DSD subtypes are likely under-diagnosed in resource-limited settings, resulting in potential underestimation of their true prevalence. Consequently, global estimates derived primarily from high-income country data may not accurately reflect the burden of disease in South Asian populations. Multicenter collaborative studies with standardized phenotyping and diagnostic approaches are essential to generate more representative data.

Systemic barriers to timely diagnosis persist in our setting, including limited access to karyotyping, hormonal profiling, and specialist multidisciplinary teams (MDTs). Structured DSD-specific Continuing Medical Education (CME) programs targeting primary care and general pediatric physicians represent a high-yield, low-cost intervention to reduce referral delays and improve early recognition of DSD phenotypes.^24^ The establishment of MDT clinics, incorporating endocrinology, pediatric surgery, clinical psychology, and genetics is essential not only for optimizing sex assignment decisions but also for addressing the substantial psychosocial burden experienced by families navigating DSD diagnoses within sociocultural environments where stigma and misinformation remain pervasive.^24^,^25^ Adoption of gender-inclusive documentation standards, as outlined in emerging health informatics frameworks, may further facilitate continuity of individualized care across clinical interfaces.^26^

### Strength

A key strength of this study is its large cohort size from a single center in a South Asian setting, providing internally consistent data on a population substantially underrepresented in global DSD literature.

### Limitations

It includes the single-center cross-sectional design, the absence of molecular genetic confirmation in the majority of cases due to resource constraints, and the lack of longitudinal follow-up data on psychosocial outcomes, gender identity evolution, and quality of life. Future research should prioritize prospective multicenter designs, I-DSD Registry linkage, and outcome-focused studies examining gender dysphoria trajectories and quality-of-life metrics across DSD subtypes in this population.

## CONCLUSION

This study highlights the clinical spectrum of DSD in a large pediatric cohort, with a predominance of 46,XY DSD and diagnostic complexity in sex chromosome DSD. These findings emphasize the importance of early recognition, careful clinical evaluation, and timely referral, particularly in resource-limited settings.

Limited access to genetic testing remains a key challenge, underscoring the need for cost-effective and locally adaptable diagnostic approaches. A multidisciplinary and individualized management strategy is essential to support appropriate clinical decision-making and reduce psychosocial burden.

While many DSD conditions can be effectively managed, outcomes vary depending on the underlying etiology and available resources. Strengthening awareness and improving access to diagnostic and supportive care are important steps toward better outcomes.

## Data Availability

The data supporting the findings of this study contain sensitive patient information and are therefore not publicly available. Access to anonymized data can be granted upon reasonable request to the corresponding author, following institutional ethical approval.
